# InCHORRRuS: Infant-Directed Communication Highlights and Organizes Repetition and Redundancy Through Rhythmic Structure

**DOI:** 10.1111/nyas.70147

**Published:** 2025-12-07

**Authors:** Camila Alviar, Warren Jones, Miriam Lense

**Affiliations:** 1 Department of Otolaryngology - Head and Neck Surgery, Vanderbilt University Medical Center, Nashville, Tennessee, USA; 2 Marcus Autism Center, Children’s Healthcare of Atlanta, Atlanta, Georgia, USA; 3 Emory University School of Medicine, Atlanta, Georgia, USA; 4 Psychology and Human Development, Vanderbilt University, Nashville, Tennessee, USA; 5 Vanderbilt Kennedy Center, Vanderbilt University Medical Center, Nashville, Tennessee, USA; 6 Hearing and Speech Sciences, Vanderbilt University, Nashville, Tennessee, USA

**Keywords:** infant-directed song, infant-directed speech, multimodal redundancy, repetition, rhythm, social development

## Abstract

Learning to successfully participate in social interactions is a monumental task for infants, whose perceptual systems are immature and communicative signals complex and hard to parse. To support their infants, caregivers naturally modify their communicative behaviors to be more repetitive, redundant, and rhythmic, thus engaging infants’ perceptual biases. In this paper, we present the InCHORRRuS framework: which considers the role of rhythm in organizing caregivers’ communicative behaviors across modalities to scaffold communication and dyadic coordination in early social interactions. We argue rhythm’s role in infant-directed (ID) communication is particularly highlighted in ID singing, in which metrically structured beat-based rhythms make the multimodal redundancy and repetition in ID communication also temporally predictable, thus “supercharging” the cues’ communicative value. Additionally, the repetition in songs, across verses and over time, offers caregivers a natural way of leveraging predictability and familiarity at the local level and at longer interactional timescales alike, increasing the impact of the enriched communicative signal. We review the current literature on timing and rhythm, redundancy, and repetition in ID signals; discuss the evidence on the confluence of redundancy and repetition in rhythmic contexts; and consider open questions and future directions our framework inspires.

## 1 | Introduction

Human communication is a complex, coordinated dance in which many different signals are woven together across time to facilitate interaction with others. To be successful participants in this dance, infants need to learn not only the specific ways the signals are combined to create meaning, but also need to become fluent in the careful timing that dictates how and when to use these behaviors to successfully shape social interactions [1]. This is a monumental task. Infants’ perceptual systems are immature, and the important elements of the communicative signals are hard to parse and uncover. In this paper, we present a framework we call InCHORRRuS (Infant-Directed Communication Highlights and Organizes Repetition and Redundancy through Rhythmic Structure) to describe how caregivers use rhythm to organize their communicative behaviors around infants in ways that facilitate infant–caregiver social interaction and communication.

In this paper, we will define rhythm in a broad sense, as the systematic patterns of temporal relationships between events [2]. This definition encompasses both the bursty hierarchical temporal relationships that characterize the rhythm of syllables, words, sentences, and turns in speech, as well as the isochronous periodicity that characterizes the metrical structure of beats in song and music [2]. Infant-directed (ID) signals tend to be more rhythmic in both senses [3, 4]. That is, they have a higher hierarchical temporal structure of events across timescales, as well as more isochronous acoustic events (see Table 1). The rhythmic patterns of child-directed speech, song, and music are all related within a culture and carry echoes of each other’s temporal structure [5]. We propose that, in both cases, the increase in acoustic structure and increased predictability extends to caregivers’ communicative behaviors across other modalities. Our argumentation here will highlight the beat-based rhythmicity of song as a natural case study that is ripe for directly exploring how the increase in isochrony and predictability in the acoustics of ID signals cascade into and provide useful structure to other behavioral modalities, making the communicative cues more accessible for the infant.

Rhythm processing is early emerging, with rhythm perception skills developing in utero and present during the neonatal period [6–8]. While other perceptual faculties may be immature at birth (e.g., visual acuity [9]) and develop over different time courses, rhythm is a salient cue for processing multimodal stimuli as it is simultaneously specified across perceptual streams (e.g., auditory and visual information) [10]. Rhythm recruits and modulates infant attention through behavioral and neural entrainment [11–15], that is, by making the temporal periodicities of the infant’s behavioral and neural signals more similar, and in some cases synchronized, to the stimulus temporal periodicities [15, 16]. Infants’ neural tracking of, and rhythmic movements to, multimodal rhythmic communication (e.g., nursery rhymes, songs) are associated with their later language development [13, 17–19]. For caregivers, rhythm provides structure to their ID communicative behaviors, allowing the multiple (redundant) meaningful information streams to come together and repeat in time in a natural and predictable way, in accordance with their infants’ attentional and cognitive biases across development.

During the first months of life, rhythm—in physiological (e.g., hormones, heartbeat), sensory (e.g., vocalizations, touch, movement), and environmental (e.g., daily light cycles and routines) signals—plays a primary role in emotional and biological regulation, as well as in early social bonding [20]. The caregiver’s physiological rhythms and sensory cues, and the environmental periodicities, organize the newborn’s early experiences and physiology by providing predictable structure to their days [20]. The rhythmicity of social interactions and of communicative signals across modalities regulates infants’ affective states, scaffolding physiological coregulation between newborns and caregivers [21]. ID singing in particular, which is often accompanied in the earliest months by rocking and affective touch, is an effective tool to modulate infants’ and caregivers’ arousal alike [22–25]. Rhythm plays a more important role in affect regulation than communication in the earlier half of the first year of life, facilitating physiological and attentional states that promote infant−caregiver social bonding and set the stage for more effective infant learning. The central role of multimodal rhythms in affect and physiological regulation in early development has been reviewed previously (see Refs. [20, 23]). Our main focus here then is on rhythm’s role in supporting infant–caregiver communication, which comes more into focus in the latter half of the first year, as infants develop new social and communicative skills such as joint attention, gestures, babbling, and then the onset of spoken words [26].

Most of the research on rhythmicity in ID communication has only considered the temporal patterns in the acoustic component of the signals. However, most interactions involving infants happen face-to-face and/or with the interacting partners in physical contact or close proximity to one another [27]. The copresence of the caregiver–infant dyad makes it possible and likely for parents to initiate and respond to their infants multimodally, incorporating also visual cues (more common in face-to-face interactions in Western cultures) and/or tactile and vestibular cues (more common of infant engagement in non-Western societies) in interaction and care routines [28, 29]. We start this paper by reviewing the temporal signatures that are typical of ID interactions and the dynamics of their acoustics. Then, we review the repetition and redundancy that characterize ID signals by looking across sensory modalities. We consider this in ID speech first, as it is the most ubiquitous (and most commonly studied) form of ID communication. We then consider the universal context of ID singing, in which beat-based rhythms provide a specific, highly predictable, isochronous structure for the repetition and redundancy in ID communication. Lastly, we discuss open questions and future research directions in the study of multimodal rhythmic cueing, including outstanding basic science questions and potential therapeutic applications. We hope to provide a framework to guide new research questions that further our understanding of the intertwining of rhythm and social communicative cues in ID communication, including to support infant communicative development and the coordination of infant–caregiver interactions.

## 2 | Temporal Signatures and Rhythmicity in ID Communication

The hallmark temporal signatures of ID communication and activities include signals that show increased rhythmicity compared to their adult counterparts in two ways (see Table 1). They are (a) generally burstier: many events clustered in close succession and separated by longer interevent intervals; and (b) more periodic, with events that occur at more regular intervals, than the adult-directed ones. The increased burstiness highlights the multilevel structure of language and social interactions across timescales [1, 30], and the increased periodicity increases the predictability of the communicative cues, favoring entrainment locally [11, 14, 31, 32].

### 2.1 | Increased Burstiness

Across contexts as varied as object play and object labeling [33, 34], parent–child physical proximity [35], infant vocal and social foraging [36], music play and singing [37], and the natural engagement/disengagement cycles of mother–infant interactions [38], infant activities tend to occur in bursty bouts. Infants’ episodes of active engagement with people and objects around them tend to be short, occur in clusters, and be separated by longer periods of lower engagement or attentional shift to other activities and objects. This kind of bursty schedule, in which the same information is encountered multiple times in spaced clusters, is thought to support learning by promoting both the short-term and long-term encoding of information [39, 40]. For example, a bursty schedule supports novel word learning better than a schedule in which words are encountered just once in close succession but not revisited [34].

**FIGURE 1 |:**
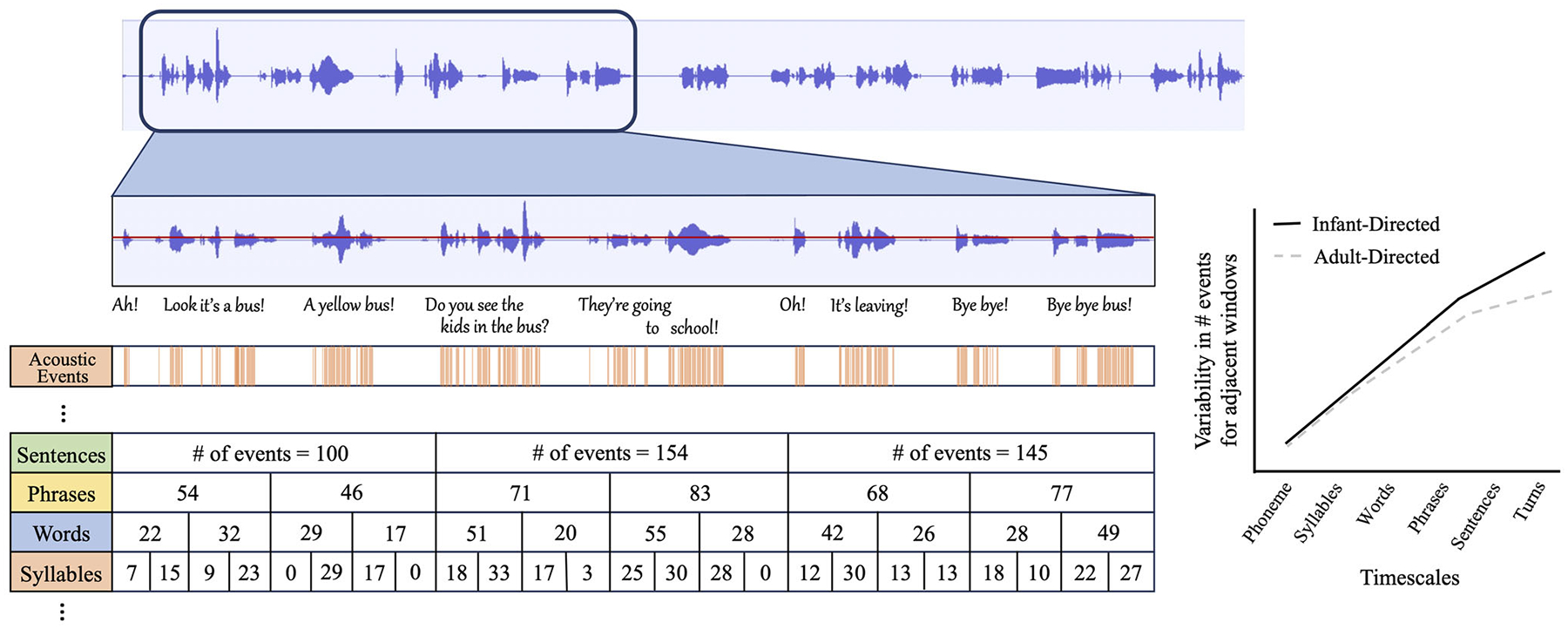
Infant-directed (ID) speech is characterized by pitch and amplitude changes, short utterances, and exaggerated pauses between them. These characteristics make it a signal that is prosodically more variable and, at the same time, burstier and more hierarchically structured than its adult-directed (AD) counterpart. At any given timescale, the increased acoustic variability in ID speech results in acoustic events (shown in orange) that are unevenly distributed over time, with periods in which many events occur in close succession, immediately followed by periods of fewer events or silence. This bursty distribution is apparent independently of the timescale under consideration (e.g., from the very short durations typical of phonemes, through increasing durations typical of syllables, words, sentences, to the longer durations of turns and topic arcs, etc.), but it gets more pronounced as we zoom out from shorter to longer timescales. More exaggerated pauses after the conclusion of a sentence than between the words in the same sentence or between phonemes of the same word create increased differences in the number of acoustic events between adjacent windows as time scale increases. (A didactic representation of this idea is shown in the event counts in the different rows in the figure, representing time scales of ~0.5 s at the syllable level, ~1 s at the word level, ~2 s at the phrase level, and ~5 s at the sentence level in our diagram). The relationship between variability and timescale follows a power law distribution (schematically represented in the figure on the right), that is, the increase in the variability of event counts between adjacent windows is proportional to the increase in window length (for a technical resource, see Ref. [30]). This stability in the relationship across timescales is more pronounced in ID (solid black line) than AD speech (dashed gray line), especially in the longer time scales, highlighting the hierarchical structure and nesting that characterize language and social interactions (see Ref. [3] for an empirical comparison of ID and AD speech and song). This results in more structured relationships between the parts of speech: acoustic events pertaining to different words cluster closer together than those for different words, and acoustic events that belong to words in the same sentence cluster closer together than those of different sentences and so on, accentuating the structured hierarchy that makes up language.

The emblematic characteristics of ID speech result in burstier distributions of sounds across timescales in the communicative signals themselves. For instance, ID speech features exaggerated pauses in between syntactic units [41, 42], extra lengthening of vowels and words in utterance-final positions [42], and decreased word duration reductions for high-frequency and highly predictable words [43, 44]. All of these, in conjunction with the more variable prosodic contours of ID speech, result in a bursty signal that brings to the forefront the hierarchical temporal structure of language, and highlights the nesting of its different timescales—phonemes that make up syllables that make up words and sentences, and so on [3, 45]. This, paradoxically, results in a speech signal that, despite being prosodically more variable, is also temporally more rigidly structured across timescales (see Figure 1). That is, a signal in which the relationships between the different parts of speech are strongly foregrounded in the acoustic organization of the signal itself.

### 2.2 | Increased Periodicity

In the acoustic domain, the increased periodicity of ID communication comes from both the characteristics of ID speech, and the ubiquitous use of ID song and rhythmic games in infant caregiving contexts across cultures [46–50]. For instance, ID speech acoustically emphasizes rhythmicity in language by highlighting temporal modulations at the scale of stress patterns as opposed to syllables, as is the case in adult-directed speech [51, 52]. ID speech also naturally shows stronger phase alignment between the amplitude modulations at the syllable level and the stress level, suggesting the syllables’ stress patterns are also more regularly timed, facilitating neural entrainment to and parsing of the speech signal [4, 51–55].

Increased periodicity also arises from the daily use of song and musical games to soothe, entertain, and communicate in infant caregiving contexts [32, 56–58]. ID song and music highlight rhythmicity in multiple ways, most notably through the use of regular, predictable beats within a metrical (hierarchically organized) structure, potentiated relative duration of stressed to unstressed syllables, and a slower tempo compared to both adult-directed song and ID speech [46, 59, 60]. Like many other activities discussed above, song and music occur in bursty bouts throughout an infant’s day [37], providing multiple opportunities for engagement with beat-based signals. Just as in speech, mothers highlight the metrical and hierarchical structure in the songs they sing by varying their prosodic emphasis while singing [3, 61]. The embedding of songs at multiple time points throughout an infant’s day, combined with the increased rhythmicity and metrical emphases within specific song interactions, contributes to a more rhythmic social soundscape for infants compared with that of adults [37, 47, 56].

## 3 | Redundancy in ID Communication

All human communicative signals, directed to adults or infants, that occur in copresent contexts involve multimodal redundancy [62–64]. Several communicative cues to emotion, syntax, semantics, and pragmatics are usually simultaneously encoded in more than one modality, and presented across the acoustics, the visuals, and/or the tactile and vestibular components of the communicative signals infants encounter. The particular set of cues used redundantly across modalities might facilitate certain communicative goals while making others more difficult. For example, using a higher pitch, increased smiling, and bouts of kissing or bouncing might all redundantly contribute to convey positive emotion to the infants, while also making vowel discrimination more difficult because of the sustained high pitch [65]. Parents naturally balance the didactic and affective functions of IDS [66] and foreground or attenuate specific cues’ occurrence and saliency depending on their communicative goals [22, 67], their infant’s developmental stage/age [61, 68], or even their culture [69]. Multimodal redundancy is useful because it captures and maintains infant attention better than cues presented unimodally [70–72], and it promotes longer and more complex play bouts with objects [73]. Additionally, it also facilitates early perceptual discrimination of useful amodal characteristics of the communicative signal that young infants are otherwise not able to discriminate unimodally [70, 71], such as differences in rhythm [10], prosody [74], affect [75], among others.

### 3.1 | Audiovisual Redundancy

Some of the redundancy infants are exposed to during language is inevitably built into the system because of the physics of producing sound with the human vocal apparatus. Lip aperture, for example, matches the acoustic contours of speech [76], and it also correlates with pitch variations in ID speech [77]. Other multimodal redundancies, such as those provided by facial expressions, although not necessary for sound production, are nevertheless present in adult-directed signals and exaggerated for infants. Head movements and body movements serve as visual correlates of acoustic prosody, matching the exaggerated pitch changes and variable acoustic contours of ID speech [78–80]. Visual and acoustic qualities of speech are also linked in the realm of emotional expression [81], with facial configurations such as smiling, for example, consistently correlating with higher pitch and formats in the voice [82].

### 3.2 | Redundancy in Action Demonstration and Gesture Use

Parent–child interactions with objects are also typically multisensory, involving visual, auditory, and haptic cues [62]. These interactions are accompanied by object-relevant words synchronized to the actions on the objects themselves [83–87], and globally correlated with the statistics of object manipulation [33, 88]. When showcasing actions for infants, parents tend to highlight action boundaries multimodally with positive facial expressions and eye contact [89, 90]. Although manual gestures are rare in ID communication, they are also more frequently encountered in conjunction with speech [62], and when present, they tend to reinforce the same information already encoded in the message rather than add to it or complement it [91].

### 3.3 | Redundancy in Touch and Vestibular Signals

The research on touch as a communicative signal is very sparse and still contextually limited, but results to date suggest that touch is used in a similar way to movement and gesture in communicative settings. That is, it is more frequently accompanied by speech than not, and more frequently aligned with words that are semantically related to it (e.g., touching body parts when talking about them) [92, 93]. Touch is the preferred mode to respond to infants’ verbal and nonverbal signals in some cultural groups (especially non-Western or rural societies) [28, 69] and thus might be a particularly salient cue for infants in those groups. The use of touch in alignment with auditory information can aid infants’ word finding and learning of statistical patterns in speech streams, suggesting touch may play a role in speech segmentation [94, 95]. Rocking and bouncing are also scarcely researched as communicative signals, but can also modulate the parsing of auditory patterns [96, 97] and tend to also be more prevalent modalities of infant engagement in non-Western societies [28].

### 3.4 | Redundancy of Ostensive Cues

In addition to reinforcing the information and offering multisensory cues to the structure of language and social action, cues across modalities can be ostensive, indicating to the infant that they are the intended recipient of the communicative signal [98–100]. Directly looking at the infant, saying their name, or touching them, are all examples of ostensive cues, which more often than not tend to be used in conjunction with one another [99]. Ostensive cues increase the infant’s attention to and cortical visual processing of objects [101], and enhance parent–child neural coupling during social interaction, which may facilitate language and social development [102].

## 4 | Repetition in ID Communication

Repetition (over time) is widely present in the linguistic, acoustic, and visual modifications of ID signals, and even in the structure of early play. Across modalities, the communicative cues and signals addressed to infants are more repetitive than those used to address older children or adults [103–108]. Repetition likely supports infant learning via associative and statistical learning mechanisms. For instance, repetition increases infants’ opportunities to engage with similar information across a variety of contexts, which facilitates more robust inferences about the statistical properties of language and the social world [109, 110]. Repetition also makes the communicative signals less complex than their adult counterparts, making infants’ hypothesis-testing about the statistics of their world more efficient [110] and reinforcing their learning [111].

### 4.1 | Repetition in Acoustic Signals

From a prosodic point of view, although ID speech is characterized by increased F0 variability and prosodic exaggeration [112, 113], such exaggeration is used in fairly consistent patterns of prosody-affect [114, 115] and prosody-syntax pairings [42, 116]. Such stability makes these consistent sound-meaning pairings repeatedly available for the infant. For instance, specific prosodic contours are associated with particular affective contexts [115, 117, 118], and the prosodic exaggeration also offers consistent cues to utterance and clause boundaries that highlight the hierarchical nature of language [116].

From a linguistic standpoint, the syntactic structures and words used when talking to infants are much simpler [106, 119] and less variable in type/complexity [108, 120, 121], which ultimately results in a more repetitive signal at various grammatical levels. Sentences are shorter [41, 107, 119, 122], include fewer clauses [116] and reduced embedding of those clauses [106], and a lower type/token ratio [119, 121, 123]. Language directed to infants also involves a higher prevalence of isolated one-word utterances [119], as well as higher contiguous verbatim repetitions (full and partial) and paraphrases of the same meaning [103, 106].

### 4.2 | Repetition in Visual Signals

Although less studied than the auditory modifications, the visual components of ID signals, observable in caregivers’ facial expressions and movements, also exhibit increased repetition and simplification across linguistic, affective, and goal-oriented action contexts. Visual prosody expressed through head nods and eyebrow movements is, like acoustic prosody, consistently used to highlight language structure visually in ID speech [78, 80, 124]. Also, paralleling the changes to spoken language during ID communication, sign language users modify the signs addressed to their infants, making them simpler, slower, exaggerated, and more repetitive [125].

In affective contexts, ID visual signals provide more direct mappings between facial expressions and affective messages. The infant-specific repertoire of facial expressions is characterized mainly by positive and comforting expressions [126], which are easily identified and classified by adults according to their addressee (e.g., adult vs. infant) and intended affective message (e.g., comforting, approving) based on pictures [126] or silent videos [127].

### 4.3 | Repetition in Action Contexts

Adults also modify their movements when showcasing actions to infants in both word-teaching contexts [83, 84] and object play [89, 128, 129]. ID action—*motionese*—involves larger and slower movements embedded in simpler and shorter action sequences, which are showcased multiple times in close succession [89, 129]. The boundaries of actions are also consistently highlighted: pauses in between action sequences are longer, and are usually accompanied by facial expressions of mock surprise [89] and eye contact [90]. Lastly, dyadic object play involves repeated turns of object exploration, action demonstration, and joint action during the same play session [128], and dyads usually return to the same few objects even when having multiple toys to choose from [33].

## 5 | Redundancy and Repetition in the Context of Beat-Based Rhythms: Song as a Predictable and Multimodally Enriched Communicative Signal

Rhythm, redundancy, and repetition are key components of ID signals, shaping the interactive contexts and communicative cues in ID speech with their combined influence. Unlike redundancy and repetition, the role of rhythmicity has not been studied in other modalities (i.e., visual, tactile, etc.) as much as in the acoustic component of the ID signals. However, early social interactions happen almost exclusively in very close proximity, either face-to-face or body-to-body, and caregivers engage their infants multimodally with acoustic, visual, tactile, and vestibular cues that naturally group together meaningfully [27, 62]. With rhythm being such a salient cue for infants, it is likely that its structuring power extends beyond acoustics to other modalities. For example, the redundancy of signals in speech suggests that the increased periodicity in speech acoustics should also be reflected in the visual and tactile cues that accompany it. The metrically organized, beat-based rhythmicity in song provides an ideal naturalistic context to study the effects of strongly predictable rhythms on the multimodal and repetitive cueing of ID communication directly.

**FIGURE 2 |:**
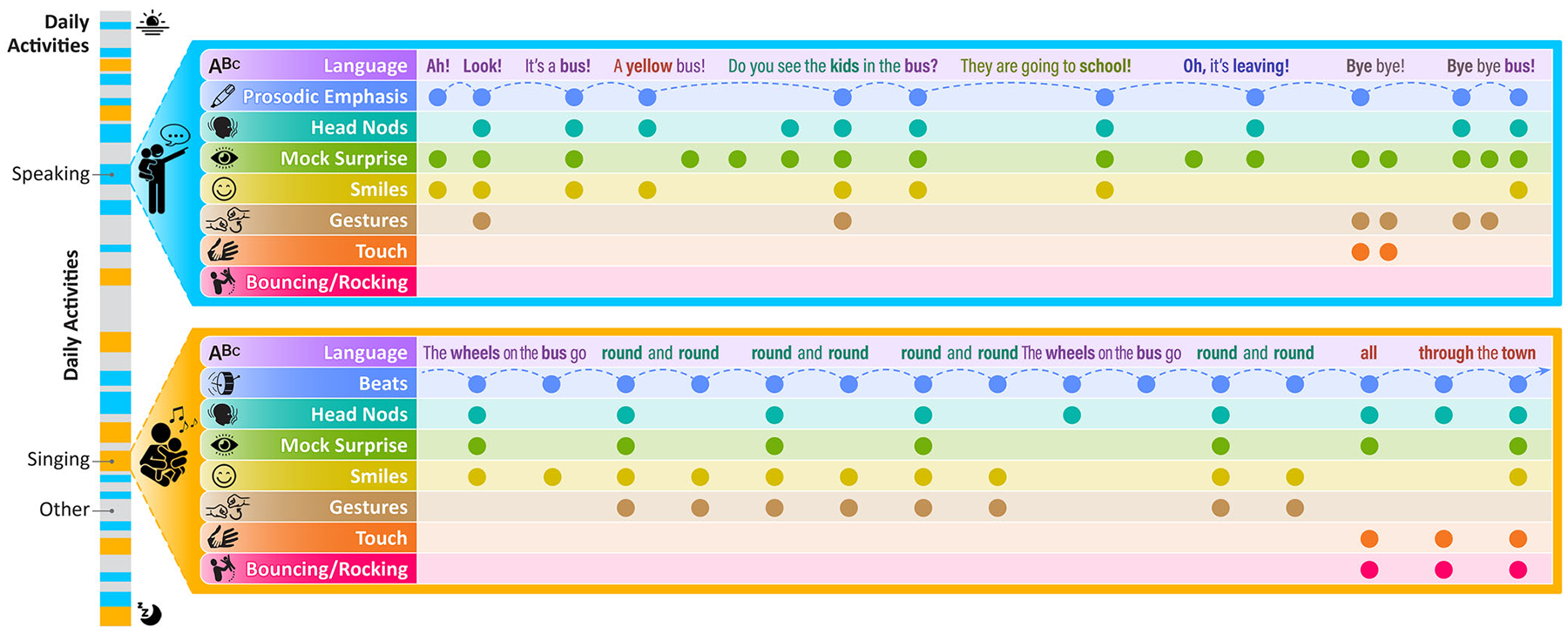
Diagrammatic representation of the InCHORRRuS framework. Caregivers speak (blue, top) and sing (orange, bottom) to their infants multiple times throughout the day in a bursty fashion (see bar on the left). Both types of communicative signals (i.e., speech, song) involve multimodal redundancy, providing meaningful cues to infants through words, head movements, facial expressions, gestures, touch, and vestibular input (notice the vertically aligned [i.e., temporally aligned] circles across rows). Both types of communicative signals also involve repetition of words and syntactic structures across time (color-coded in the “Language” row of each signal), as well as repetition of sets of multimodal cues during and across interactions (notice the combinations of cues that repeat over time). In both speech and song, caregivers provide infants with exaggerated and redundant cues that make it easier to identify interactive, communicative, and linguistic information. In song (orange rectangle, bottom), the isochronous beat-based rhythmicity (blue “Beats” row) structures the repetitive and redundant cues, facilitating prediction of when they will occur in time. Rhythmic structuring facilitates infants’ attention to and processing of the rich set of communicative cues, supercharging their communicative value.

We propose that the beat-based isochronous rhythmicity of ID song naturally creates a context in which the repetition and multimodal redundancy of ID signals become particularly *predictable and temporally salient* for the infant as they align synchronously or coordinate contingently with the rhythmic structure of the song. Such confluence makes ID song a *naturally enriched sociocommunicative signal* that offers *optimal learning and coordination opportunities* for the infant and the infant–caregiver dyad. Figure 2 presents a visual diagram of this proposal, highlighting the predictability that beat-based rhythms add to both the repetitiveness and multimodal redundancy in ID communication.

ID song exhibits substantial multimodal redundancy. For example, caregivers smile more when singing versus speaking to their infant, and singing involves increased facial movements, gestures, and overall audiovisual synchrony compared to speech [61, 130, 131]. The isochronous rhythmicity in song adds an organizing layer to the timing of these multimodal cues (see Figure 2 for a schematic example). When singing, caregivers are more likely to decrease their neutral affect and increase their eye expressiveness and social gaze, opening their eyes wider and inhibiting blinking, in alignment with the beats than in between the beats [11]. Similarly, mothers synchronize their head nods, body bouncing, rocking, swaying, tickling, and toy bouncing with the beats of their ID singing [61].

Rhythmicity also organizes the repetition of ID signals in specific ways (see the “language” row in Figure 2 for an example; notice the pattern in which words and phrases repeat and new words are introduced). Although ID speech is already grammatically and morphologically more repetitive than adult-directed speech (as discussed in section 4), the language in ID songs is more so, even when compared with children’s stories or adult songs [132]. Beyond the increased repetition, however, the balance of repeated versus new information occurs in an oscillatory and rhythmic pattern in songs, making the flow of information optimal for infants’ attention and learning mechanisms [132], and perhaps highlighting the linguistic repetition even more. Additionally, many infant songs and rhythmic games are accompanied by specific gestures and hand motions that occur in time with the rhythm and align with specific words or musical verses [133]. This offers consistent multimodal pairings of gestures with relevant words and acoustic cues that repeat rhythmically throughout the song and each time it is performed (e.g., rolling the arms around each other while singing “round and round” during “Wheels on the Bus”). Such a confluence of repetition and multimodality in time with the rhythmic structure makes ID song an enhanced social signal that repeatedly scaffolds and modulates infants’ attention to many sociocommunicative cues at once [11].

**FIGURE 3 |:**
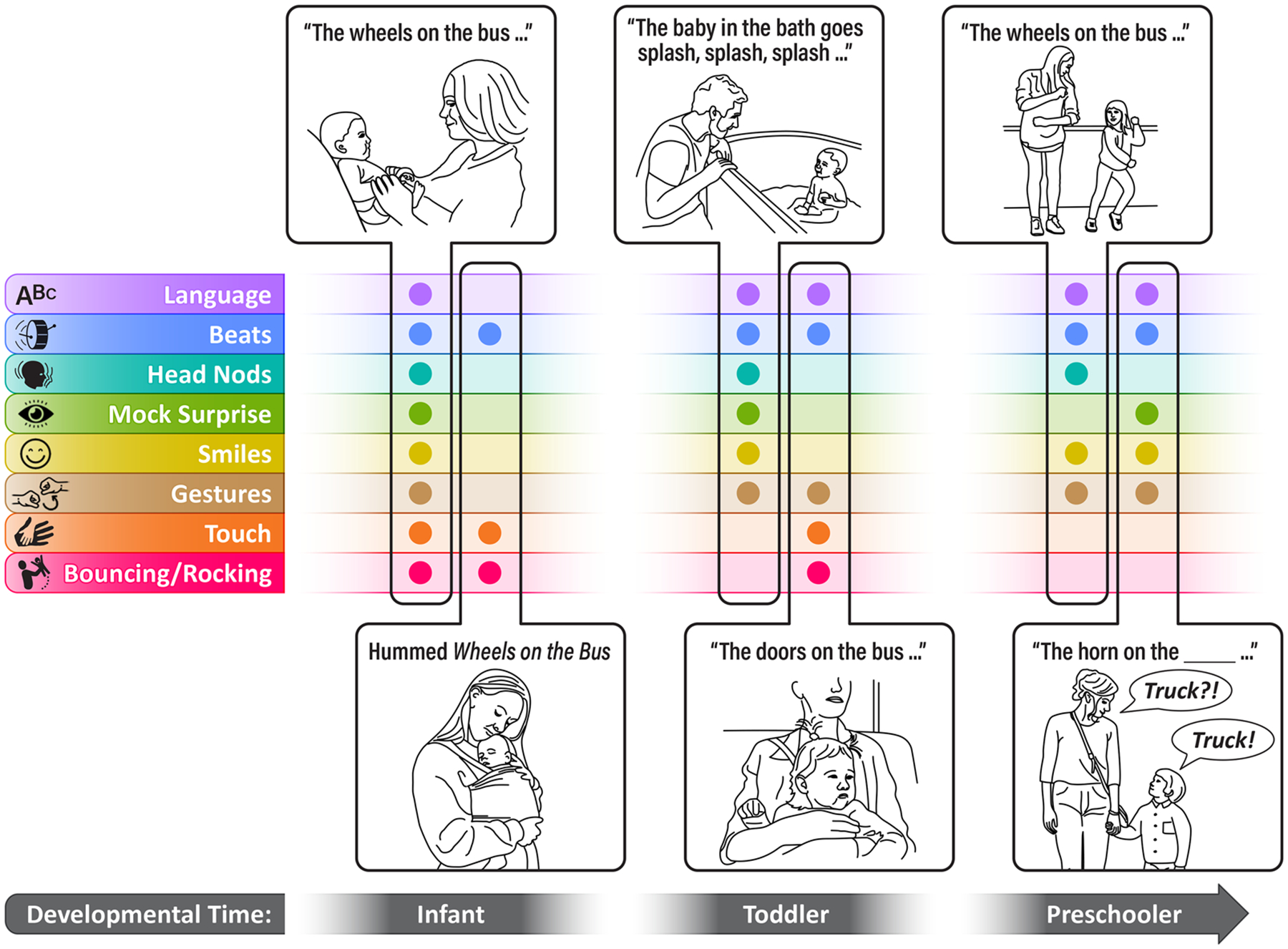
The sets of multimodal cues that caregivers highlight within the rhythmic structure of a song are variable across repeated verses and performances depending on whether their infant can see them, their interactional goals, and their child’s current needs and skillset, among other constraints. One can imagine a caregiver engaging in a full-on performance by smiling, nodding, gesturing, and bouncing their infants on their knees as they sing playfully (first vignette from the left), humming the verses of their child’s favorite song while patting and rocking them to sleep (second vignette from the left), or even changing the lyrics to match the activities at hand during bath time, for example (third vignette from the left). Such flexibility and novelty in the communicative cueing are, at the same time, made familiar and predictable by the common thread of the rhythmic and melodic structure of the songs, engaging infants’ preference for novelty and familiarity at the same time. The repetition of favorite songs throughout development additionally scaffolds interpersonal coordination across longer timescales by offering predictable interactive structures that facilitate infants’ social participation as they gradually gain more skills: from smiling back and energetically moving as their caregivers sing in the first months of life; to gesturing along, vocalizing/filling in key words on the beat, and bouncing up and down in late infancy; to singing and dancing along, pantomiming actions, and choosing the next verse in toddlerhood and preschool (last two vignettes).

Despite song naturally being a routinized performance, it is not rote. Caregivers vary the acoustic parameters (e.g., tempo and pitch) as they repeatedly perform a given song for their infants across time and settings [134]. Similarly, the specific multimodal cues that align with the rhythm are not always the same across repeated renditions and are instead likely to respond to the infant’s developmental needs, skills, and feedback; the situational constraints; and the dyads’ individual and cultural patterns of parental responsiveness (see Figures 2 and 3). For instance, when wearing their infant on their body, the caregiver might use touch and vestibular cues in synchrony with the rhythmic structure more, while prioritizing instead head nods and facial expressions when in the infant’s field of view during feeding or face-to-face play. This flexibility within structure allows caregivers to present their infants with novel and rich sociocommunicative information within a familiar and predictable context, engaging their preferences for novelty [135] and familiarity [134] at the same time. The strong predictability of actions, language, and routines in song may also support the development of statistical learning skills in infants [136].

Beyond supporting repeated attention to and practice with specific communicative cues, ID song also scaffolds infants’ social development and active participation more broadly by repeatedly engaging them with familiar interactive structures that facilitate dyadic coordination across modalities and time scales [1, 137]. The repetition and stability of the rhythmic structure across verses of a song offer infants repeated practice with the communicative cues and the interactive structures of social interaction at short timescales. And, infants’ preference for a small set of familiar songs [134, 138], repeatedly favored in their caregiver’s repertoire [56, 139], extends such practice to longer developmental timescales (see Figure 3). The stability of the song repertoire throughout developmental stages scaffolds infants’ sociocommunicative development by providing a familiar interactive structure to organize their participation in social musical experiences as their skills grow: from watching, smiling, and vigorously moving in early infancy; to gesturing, dancing, and vocalizing to fill in rhythmically salient words/sounds between the first and second year of life; to singing along with their caregivers and expanding their song-associated gesture repertoire during toddlerhood; to choosing the next verse and making up funny lyrics to familiar melodies in early preschool [140–143].

## 6 | Open Questions and Future Directions

### 6.1 | Moderators of the Effects of Rhythmic Structure on the Redundancy and Repetition of the Signals

The specifics of how, when, and which cues are coordinated with the rhythmic structure in ID song and speech, and how such coordination changes over time to support parent–child communication and infant development, are still understudied and an area of future research. So far, there is evidence to suggest that caregivers’ communicative cueing is sensitive to variables such as infant feedback, caregivers’ communicative intentions, and infant development/age. Caregiver cueing is dynamically shaped by infants’ real-time feedback and behaviors (see also Ref. [144]). The acoustic cues in caregivers’ songs differ when caregivers sing to their infant versus when they pretend to sing to them in their absence, or when caregivers can see their infants as they sing versus when their view of their infant is being blocked [131, 145]. Caregivers’ acoustics [22, 145–147] and movements [22] also differ for play and lullaby versions of the same song, suggesting that cueing is also shaped by caregivers’ interactional goals. Acoustic and multimodal cueing in song is also sensitive to infant developmental needs and skills, as reflected by changes in cueing related to the infant’s age [61, 148]. For instance, mothers highlight the middle and final beats of songs more for their younger infants (3−4 months) and use more head nods, and infant touching and bouncing to do so, while marking these metrical differences less strongly for their older infants (7−8 months) and using mostly toy bouncing and tickling [61]. Mothers further modify their acoustics for their preschoolers, singing in a lower pitch and enunciating more clearly in comparison to when they sing to their infant [149]. Compared to mothers of typically developing infants, mothers of infants with Down Syndrome sing with a higher pitch, more tonal variability, and take longer to reach a clear rhythmic structure in their songs [146].

The research on moderators of caregiver cueing so far has mostly concerned the acoustics of the signals. However, it is likely that the same variables (i.e., infant feedback, caregiver intentions, and infant developmental needs) also modulate the use of communicative cues in other modalities, and the amount and timing of their repetition and redundancy in song or speech. For example, in Western cultures in which face-to-face interactions are preferred, it may be that facial expressions and visual prosody in the face are more aligned with rhythm early in development, as infants are mostly looking at their parents’ faces, and then such alignment transitions to the hands and gestures as infants spend more time sitting upright and attending to hands [150]. Multimodal cueing in song (e.g., how much caregivers smile or how much they pat or bounce their infant) may also dynamically adapt to infant feedback in real-time as it does in ID speech [144]. Infant attention, engagement, affective states, and behaviors shape caregivers’ responses to them, and are in turn shaped by the caregiver in a social-feedback loop [139, 151–153]. For example, caregivers tend to respond rhythmically to their infants’ rhythmic movements and rhythmic vocalizations [154, 155], and their babies in turn tend to move more, and more rhythmically, during rhythmic stimulation [13, 61]. These rhythmic feedback loops between parent and child are associated with child language development [154]. The specifics on how infants’ behaviors might start, maintain, and/or dynamically modify their caregivers’ rhythmic cueing across different modalities are an area of future research.

Cultural and individual differences may also play a role in the way the multimodal redundancy and repetition of cues are interwoven within the rhythmicity of ID song and speech. The frequency of use of certain cues may change given cultural differences such as the use of facial expressions to reveal emotions [156, 157], the rate and quality of gesture use [158], the degree to which adults modify their cueing for ID communication [103, 117], and the variability in infant wearing and other caregiving practices that may make visual versus tactile or vestibular cues more important in ID communication (e.g., facial expressions vs. bouncing or patting) [28]. With respect to individual differences, mothers’ social motivation, for example, increases the frequency of their engagement in ID singing [159], and might also modulate how expressive parental singing is across different modalities.

Lastly, it is also an open question how precisely the cues in other modalities might synchronize with the beats during singing. While punctate cues of shorter duration, like head nods, pats, and bounces, might synchronize precisely in-phase with the beats, the relationship with cues of longer duration, such as smiles or gestures, might be one of increased likelihood on versus off beat or with regard to the metrical hierarchy. It is possible that certain elements of the more continuous cues when broken down relate in more precise ways to the rhythmic structure. For example, it is an empirical question how the onset versus the point of maximum extension versus the termination of a gesture or a smile are timed with respect to the beats. And whether the duration of a gesture or a smile, or even the force or displacement of a bounce, could also be used to encode larger groupings in the metrical structure of the songs (as gestures do for intonational units in adult-directed speech [160]). It is likely that such timing relationships are variable and change with infants’ growing language and social skills [61].

### 6.2 | Possible Therapeutic Applications of the Enriched Rhythmic Signal

For all the richness of the sociocommunicative cueing that caregivers provide for infants when singing, it is perhaps surprising that producing such a complex signal is relatively intuitive for most caregivers. In general, parents do not need to be taught how to modify their voices and movements to best engage their infants [99]. The fact that caregivers across cultures have infant-specific songs whose communicative intentions adults from other cultures can easily recognize in unfamiliar languages [46, 161, 162] suggests that at least some of the modifications of ID song are common across cultures [46] and might be part of the “natural pedagogy” of infant care (cf. Refs. [99, 163]). The confluence of redundancy, repetition, and rhythmic predictability ID song facilitates might offer an easy and universal way for caregivers to engage their infants in high-quality interactions that promote bonding [23, 31, 164] and highlight the timing of the social world at many scales [1, 3]. It is an open question whether producing high-quality signals might be easier in a singing than a speech context as a result of the added routinized components and rhythmic structure. This future line of inquiry can be particularly promising for populations who face additional difficulties creating or maintaining engaging ID signals, as a result of decreased mental health (e.g., depression [165–167]) or disrupted social feedback from their children (e.g., in autism [168]). ID song supports affect regulation in infants and parents alike [22, 138, 169], and can be an intrinsically rewarding experience for the caregiver and child [170], which could promote the creation of more frequent opportunities for positive interactions. This, in addition to song being a routinized activity, and in conjunction with the strong entrainment mechanisms [11, 171, 172], might scaffold and support the occurrence, timing, and receipt of quality multimodal cueing. Future research could examine this possibility and investigate how singing could be used as a tool to naturally support parents who have difficulty creating or maintaining high-quality interactions with their children.

## 7 | Conclusion

Rhythm is a powerful tool to support infant sociocommunicative development. As reviewed by the InCHORRRuS framework presented here, rhythm naturally offers structure to the multimodally redundant and repetitive cues in the caregiver’s communicative signals, supercharging their saliency by increasing their predictability. It also naturally modulates infant attention, guiding it to those important times in the communicative signal, and scaffolding caregiver–infant coordination. With its metrically structured, beat-based rhythmicity, and its flexible embedding throughout daily routines and across caregiving activities, ID singing may be an especially effective context to support and study caregiver communication, and infant language learning and interactive participation.

## Figures and Tables

**TABLE 1 |:** Types of rhythm in ID signals.

Rhythm: Systematic pattern of events in time [2]	Hierarchical temporal structure	Periodicity
**ID speech**	Increased burstiness with variability that scales across timescales more strongly than in adult-directed speech [3, 45] (Figure 1). The prosodic variability and exaggerated pauses of the ID register produce clearer boundaries between parts of speech at different timescales (e.g., sounds and syllables from the same word occur closer together than those of different words, and sounds from words in the same sentence are acoustically grouped closer together than sounds from different sentences), highlighting the hierarchical nestedness of language.	Although speech does not exhibit music’s beat-based periodicity, ID speech’s spectral and acoustic energy modulations emphasize the stress patterns in speech, and the syllables are timed to occur both more evenly and in alignment with the stress patterns, producing a more periodic signal than adult-directed speech [4, 50–52].
**ID song**	Clear metrical structure. The beats in ID song are organized hierarchically in a metrical structure of alternating strong and weak beats [2,173] (e.g., the simple 4/4 pattern of most Western children’s music, which remains stable throughout the song), and is often acoustically highlighted.	Beat-based rhythmicity with acoustic events organized around a central underlying pulse that isochronously repeats [2]. The acoustic signal is generally prominent on the beat in children’s songs, though beats can be inferred even without co-occurring acoustic events (e.g., when a caregiver pauses their singing vocalization to breathe).

## References

[1] Rączaszek-Leonardi J, Główka K, Nomikou I, , “Time-to-Smile, Time-to-Speak, Time-to-Resolve: Timescales for Shaping Engagement in Language,” Language Sciences 93 (2022): 101495, 10.1016/j.langsci.2022.101495.

[2] Patel AD, “Rhythm,” Music, Language, and the Brain (Oxford University Press, 2007), 10.1093/acprof:oso/9780195123753.003.0003.

[3] Falk S and Kello CT, “Hierarchical Organization in the Temporal Structure of Infant-Direct Speech and Song,” Cognition 163 (2017): 80–86, 10.1016/j.cognition.2017.02.017. 28292666

[4] Leong V, Kalashnikova M, Burnham D, , “Infant-Directed Speech Enhances Temporal Rhythmic Structure in the Envelope,” Interspeech 2014 (2014), 2563–2567, 10.21437/Interspeech.2014-549.

[5] Hannon EE, Lévêque Y, Nave KM, , “Exaggeration of Language-Specific Rhythms in English and French Children’s Songs,” Frontiers in Psychology 7 (2016): 939. 27445907 10.3389/fpsyg.2016.00939 PMC4914820

[6] Arioli M, Silvestri V, Giannì ML, , “The Impact of Rhythm on Visual Attention Disengagement in Newborns and 2-Month-Old Infants,” Cognition 257 (2025): 106077, 10.1016/j.cognition.2025.106077. 39908636

[7] Bianco R, Tóth B, and Bigand F, Human Newborns Form Musical Predictions Based on Rhythmic but Not Melodic Structure. (2025), 10.1101/2025.02.19.639016. PMC12875487 41642755

[8] Saadatmehr B, Edalati M, Wallois F, , “Auditory Rhythm Encoding During the Last Trimester of Human Gestation: From Tracking the Basic Beat to Tracking Hierarchical Nested Temporal Structures,” Journal of Neuroscience 45 (2024): e0398242024, 10.1523/JNEUROSCI.0398-24.2024. PMC11756625 39715688

[9] Kiorpes L, “The Puzzle of Visual Development: Behavior and Neural Limits,” Journal of Neuroscience 36 (2016): 11384–11393, 10.1523/JNEUROSCI.2937-16.2016. 27911740 PMC5125205

[10] Lewkowicz DJ, “Learning and Discrimination of Audiovisual Events in Human Infants: The Hierarchical Relation Between Intersensory Temporal Synchrony and Rhythmic Pattern Cues,” Developmental Psychology 39 (2003): 795–804, 10.1037/0012-1649.39.5.795. 12952394

[11] Lense M, Shultz S, Astésano C, , “Music of Infant-Directed Singing Entrains Infants’ Social Visual Behavior,” Proceedings of the National Academy of Sciences 119 (2022): e2116967119, 10.1073/pnas.2116967119. PMC9659341 36322755

[12] Mandke K and Rocha S, Neural and Behavioural Rhythmic Tracking During Language Acquisition: The Story so Far. (2023), 10.31234/osf.io/6d3cq.

[13] Nguyen T, Reisner S, Lueger A, , Sing to Me, Baby: Infants Show Neural Tracking and Rhythmic Movements to Live and Dynamic Maternal Singing. (2023), 10.1101/2023.02.28.530310. PMC10618693 37879243

[14] Wass S, Contingencies and Periodicities: Two Types of Predictability in Children’s Real-World Environment That Might Influence the Executive Control of Attention. 2024, 10.31234/osf.io/z6rjc.

[15] Nencheva ML and Lew-Williams C, “Understanding Why Infant-Directed Speech Supports Learning: A Dynamic Attention Perspective,” Developmental Review 66 (2022): 101047, 10.1016/j.dr.2022.101047.

[16] Clayton M, “What Is Entrainment? Definition and Applications in Musical Research,” Empirical Musicology Review 7 (2012): 49–56, 10.18061/1811/52979.

[17] Attaheri A, Choisdealbha ÁN, and Rocha S, Infant Low-Frequency EEG Cortical Power, Cortical Tracking and Phase-Amplitude Coupling Predicts Language a Year Later. (2024), 10.1101/2022.11.02.514963. PMC11620356 39636849

[18] Ní Choisdealbha Á, Attaheri A, Rocha S, , “Neural Phase Angle From Two Months When Tracking Speech and Non-Speech Rhythm Linked to Language Performance From 12 to 24 Months,” Brain and Language 243 (2023): 105301, 10.1016/j.bandl.2023.105301. 37399686

[19] Menn KH, Ward EK, Braukmann R, , “Neural Tracking in Infancy Predicts Language Development in Children With and Without Family History of Autism,” Neurobiology of Language 3 (2022): 495–514, 10.1162/nol_a_00074. 37216063 PMC10158647

[20] Bobin-Bègue A, “Rhythms in Early Development,” in Early Interaction and Developmental Psychopathology: Volume I: Infancy, ed. Apter G, Devouche E, and Gratier M (Springer International Publishing, 2019), 55–86, 10.1007/978-3-030-04769-6_4.

[21] Feldman R, Magori-Cohen R, Galili G, , “Mother and Infant Coordinate Heart Rhythms Through Episodes of Interaction Synchrony,” Infant Behavior and Development 34 (2011): 569–577, 10.1016/j.infbeh.2011.06.008. 21767879

[22] Cirelli LK, Jurewicz ZB, and Trehub SE, “Effects of Maternal Singing Style on Mother–Infant Arousal and Behavior,” Journal of Cognitive Neuroscience 32 (2020): 1213–1220, 10.1162/jocn_a_01402. 30912725

[23] Sharman KM, Meissel K, and Henderson AME, “The Effects of Live Parental Infant-Directed Singing on Infants, Parents, and the Parent-Infant Dyad: A Systematic Review of the Literature,” Infant Behavior and Development 72 (2023): 101859, 10.1016/j.infbeh.2023.101859. 37343492

[24] Corbeil M, Trehub SE, and Peretz I, “Singing Delays the Onset of Infant Distress,” Infancy 21 (2016): 373–391, 10.1111/infa.12114.

[25] Shenfield T, Trehub SE, and Nakata T, “Maternal Singing Modulates Infant Arousal,” Psychology of Music 31 (2003): 365–375, 10.1177/03057356030314002.

[26] Gratier M and Devouche E, “The Development of Infant Participation in Communication,” in Early Vocal Contact and Preterm Infant Brain Development: Bridging the Gaps Between Research and Practice, ed. Filippa M, Kuhn P, and Westrup B (Springer International Publishing, 2017), 55–69, 10.1007/978-3-319-65077-7_4.

[27] Tamis-LeMonda CS and Masek LR, “Embodied and Embedded Learning: Child, Caregiver, and Context,” Current Directions in Psychological Science 32 (2023): 369–378, 10.1177/09637214231178731. 41536421 PMC12799248

[28] Keller H, Lohaus A, and Kuensemueller P, “The Bio-Culture of Parenting: Evidence From Five Cultural Communities,” Parenting: Science and Practice 4 (2004): 25–50, 10.1207/s15327922par0401_2.

[29] Kärtner J, Keller H, Lamm B, , “Similarities and Differences in Contingency Experiences of 3-Month-Olds Across Sociocultural Contexts,” Infant Behavior and Development 31 (2008): 488–500, 10.1016/j.infbeh.2008.01.001. 18272226

[30] Kello CT, Dalla Bella S, Médé B, , “Hierarchical Temporal Structure in Music, Speech and Animal Vocalizations: Jazz Is Like a Conversation, Humpbacks Sing Like Hermit Thrushes,” Journal of the Royal Society Interface 14 (2017): 20170231, 10.1098/rsif.2017.0231. 29021158 PMC5665819

[31] Markova G, Nguyen T, and Hoehl S, “Neurobehavioral Interpersonal Synchrony in Early Development: The Role of Interactional Rhythms,” Frontiers in Psychology 10 (2019): 2078. 31620046 10.3389/fpsyg.2019.02078 PMC6759699

[32] Nguyen T, Flaten E, and Trainor L, “Early Social Communication Through Music: State of the Art and Future Perspectives,” Developmental Cognitive Neuroscience 63 (2023): 121079, 10.31234/osf.io/j5g69. PMC10407289 37515832

[33] Karmazyn-Raz H and Smith LB, “Discourse With Few Words: Coherence Statistics, Parent-Infant Actions on Objects, and Object Names,” Language Acquisition 30 (2023): 211–229, 10.1080/10489223.2022.2054342. 37736139 PMC10513098

[34] Slone LK, Abney DH, Smith LB, , “The Temporal Structure of Parent Talk to Toddlers About Objects,” Cognition 230 (2023): 105266, 10.1016/j.cognition.2022.105266. 36116401 PMC13244219

[35] Suarez-Rivera C, Pinheiro-Mehta N, and Tamis-LeMonda CS, “Within Arms Reach: Physical Proximity Shapes Mother-Infant Language Exchanges in Real-Time,” Developmental Cognitive Neuroscience 64 (2023): 101298, 10.1016/j.dcn.2023.101298. 37774641 PMC10534257

[36] Warlaumont AS, Sobowale K, and Fausey CM, “Daylong Mobile Audio Recordings Reveal Multitimescale Dynamics in Infants’ Vocal Productions and Auditory Experiences,” Current Directions in Psychological Science 31 (2022): 12–19, 10.1177/09637214211058166. 35707791 PMC9197087

[37] Mendoza JK and Fausey CM, “Everyday Parameters for Episode-to-Episode Dynamics in the Daily Music of Infancy,” Cognitive Science 46 (2022): e13178, 10.1111/cogs.13178. 35938844 PMC9542518

[38] Brazelton TB, Tronick E, Adamson L, , “Early Mother-Infant Reciprocity,” Ciba Foundation Symposium (1975), 10.1002/9780470720158.ch9. 1045978

[39] Vlach HA, Sandhofer CM, and Kornell N, “The Spacing Effect in Children’s Memory and Category Induction,” Cognition 109 (2008): 163–167, 10.1016/j.cognition.2008.07.013. 18835602

[40] Vlach HA, Sandhofer CM, and Bjork RA, “Equal Spacing and Expanding Schedules in Children’s Categorization and Generalization,” Journal of Experimental Child Psychology 123 (2014): 129–137, 10.1016/j.jecp.2014.01.004. 24613074 PMC3995866

[41] Fernald A, Taeschner T, Dunn J, , “A Cross-Language Study of Prosodic Modifications in Mothers’ and Fathers’ Speech to Preverbal Infants*,” Journal of Child Language 16 (1989): 477–501, 10.1017/S0305000900010679. 2808569

[42] Fisher C and Tokura H, “Acoustic Cues to Grammatical Structure in Infant-Directed Speech: Cross-Linguistic Evidence,” Child Development 67 (1996): 3192–3218, 10.2307/1131774. 9071777

[43] Pate JK and Goldwater S, “Talkers Account for Listener and Channel Characteristics to Communicate Efficiently,” Journal of Memory and Language 78 (2015): 1–17, 10.1016/j.jml.2014.10.003.

[44] Tippenhauer N, Fourakis ER, Watson DG, , “The Scope of Audience Design in Child-Directed Speech: Parents’ Tailoring of Word Lengths for Adult Versus Child Listeners,” Journal of Experimental Psychology: Learning, Memory, and Cognition 46 (2020): 2163–2178, 10.1037/xlm0000939. 32700933 PMC8522436

[45] Boorom O, Alviar C, Zhang Y, , “Child Language and Autism Diagnosis Impact Hierarchical Temporal Structure of Parent-Child Vocal Interactions in Early Childhood,” Autism Research 15 (2022): 2099–2111, 10.1002/aur.2804. 36056678 PMC9995224

[46] Hilton CB, Moser CJ, and Bertolo M, “Acoustic Regularities in Infant-Directed Speech and Song Across Cultures,” Nature Human Behaviour 6 (2022): 1545–1556, 10.1101/2020.04.09.032995. PMC10101735 35851843

[47] Hippe L, Hennessy V, Ramirez NF, , “Comparison of Speech and Music Input in North American Infants’ Home Environment Over the First 2 Years of Life,” Developmental Science 27 (2024): e13528, 10.1111/desc.13528. 38770599 PMC12313026

[48] Mehr SA, Singh M, Knox D, , “Universality and Diversity in Human Song,” Science 366 (2019): eaax0868, 10.1126/science.aax0868. PMC7001657 31753969

[49] Yan R, Jessani G, Spelke ES, , “Across Demographics and Recent History, Most Parents Sing to Their Infants and Toddlers Daily,” Philosophical Transactions of the Royal Society of London Series B: Biological Sciences 376 (2021): 20210089, 10.1098/rstb.2021.0089. 34719251 PMC8558774

[50] Payne E, Post B, and Astruc L, Rhythmic Modification in Child Directed Speech (2015).

[51] Leong V, Kalashnikova M, and Burnham D, “The Temporal Modulation Structure of Infant-Directed Speech,” Open Mind 1 (2017): 78–90, 10.1162/OPMI_a_00008.

[52] Perez-Navarro J, Lallier M, Clark C, , “Local Temporal Regularities in Child-Directed Speech in Spanish,” Journal of Speech, Language, and Hearing Research 65 (2022): 3776–3788, 10.1044/2022_JSLHR-22-00111. 36194778

[53] Attaheri A, Choisdealbha ÁN, Di Liberto GM, , “Delta- and Theta-Band Cortical Tracking and Phase-Amplitude Coupling to Sung Speech by Infants,” Neuroimage 247 (2022): 118698, 10.1016/j.neuroimage.2021.118698. 34798233

[54] Menn KH, Michel C, Meyer L, , “Natural Infant-Directed Speech Facilitates Neural Tracking of Prosody,” Neuroimage 251 (2022): 118991, 10.1016/j.neuroimage.2022.118991. 35158023

[55] Suppanen E, Huotilainen M, and Ylinen S, “Rhythmic Structure Facilitates Learning From Auditory Input in Newborn Infants,” Infant Behavior and Development 57 (2019): 101346, 10.1016/j.infbeh.2019.101346. 31491617

[56] Mendoza JK and Fausey CM, “Everyday Music in Infancy,” Developmental Science 24 (2021): e13122, 10.1111/desc.13122. 34170059 PMC8596421

[57] Steinberg S, Shivers CM, Liu T, , “Survey of the Home Music Environment of Children With Various Developmental Profiles,” Journal of Applied Developmental Psychology 75 (2021): 101296, 10.1016/j.appdev.2021.101296. 34737486 PMC8562654

[58] Trehub SE and Trainor L, “Singing to Infants: Lullabies and Play Songs,” Advances in Infancy Research 12 (1998): 43–78.

[59] Albouy P, Mehr SA, Hoyer RS, , “Spectro-Temporal Acoustical Markers Differentiate Speech From Song Across Cultures,” Nature Communications 15 (2024): 4835, 10.1038/s41467-024-49040-3. PMC11156671 38844457

[60] Yu CY, Cabildo A, Grahn JA, , “Perceived Rhythmic Regularity Is Greater for Song Than Speech: Examining Acoustic Correlates of Rhythmic Regularity in Speech and Song,” Frontiers in Psychology 14 (2023): 1167003. 37303916 10.3389/fpsyg.2023.1167003 PMC10250601

[61] Longhi E, “Songese’: Maternal Structuring of Musical Interaction With Infants,” Psychology of Music 37 (2009): 195–213, 10.1177/0305735608097042.

[62] Kosie JE and Lew-Williams C, “Infant-Directed Communication: Examining the Many Dimensions of Everyday Caregiver-Infant Interactions,” Developmental Science 27 (2024): e13515, 10.1111/desc.13515. 38618899 PMC11333185

[63] Levinson SC and Holler J, “The Origin of Human Multi-Modal Communication,” Philosophical Transactions of the Royal Society B: Biological Sciences 369 (2014): 20130302, 10.1098/rstb.2013.0302. PMC4123681 25092670

[64] Suarez-Rivera C, Schatz JL, Herzberg O, , “Joint Engagement in the Home Environment Is Frequent, Multimodal, Timely, and Structured,” Infancy 27 (2022): 232–254, 10.1111/infa.12446. 34990043

[65] Trainor LJ and Desjardins RN, “Pitch Characteristics of Infant-Directed Speech Affect Infants’ Ability to Discriminate Vowels,” Psychonomic Bulletin & Review 9 (2002): 335–340, 10.3758/BF03196290. 12120797

[66] Kempe V, Schaeffler S, and Thoresen JC, “Prosodic Disambiguation in Child-Directed Speech,” Journal of Memory and Language 62 (2010): 204–225, 10.1016/j.jml.2009.11.006.

[67] Bryant GA and Barrett HC, “Recognizing Intentions in Infant-Directed Speech: Evidence for Universals,” Psychological Science 18 (2007): 746–751, 10.1111/j.1467-9280.2007.01970.x. 17680948

[68] Rosslund A, Mayor J, Mundry R, , “A Longitudinal Investigation of the Acoustic Properties of Infant-Directed Speech From 6 to 18 Months,” Royal Society Open Science 11 (2024): 240572, 10.1098/rsos.240572. 39525362 PMC11544372

[69] Kärtner J, Keller H, and Yovsi RD, “Mother–Infant Interaction During the First 3 Months: The Emergence of Culture-Specific Contingency Patterns,” Child Development 81 (2010): 540–554, 10.1111/j.1467-8624.2009.01414.x. 20438459

[70] Bahrick LE, Lickliter R, and Flom R, “Intersensory Redundancy Guides the Development of Selective Attention, Perception, and Cognition in Infancy,” Current Directions in Psychological Science 13 (2004): 99–102, 10.1111/j.0963-7214.2004.00283.x.

[71] Bahrick LE and Lickliter R, “Intersensory Redundancy Guides Attentional Selectivity and Perceptual Learning in Infancy,” Developmental Psychology 36 (2000): 190–201, 10.1037/0012-1649.36.2.190. 10749076 PMC2704001

[72] Suarez-Rivera C, Smith LB, and Yu C, “Multimodal Parent Behaviors Within Joint Attention Support Sustained Attention in Infants,” Developmental Psychology 55 (2019): 96–109, 10.1037/dev0000628. 30489136 PMC6296904

[73] Schatz JL, Suarez-Rivera C, Kaplan BE, , “Infants’ Object Interactions Are Long and Complex During Everyday Joint Engagement,” Developmental Science 25 (2022): e13239, 10.1111/desc.13239. 35150058 PMC10184133

[74] Bahrick LE, McNew ME, Pruden SM, , “Intersensory Redundancy Promotes Infant Detection of Prosody in Infant-Directed Speech,” Journal of Experimental Child Psychology 183 (2019): 295–309, 10.1016/j.jecp.2019.02.008. 30954804 PMC6980335

[75] Flom R and Bahrick LE, “The Development of Infant Discrimination of Affect in Multimodal and Unimodal Stimulation: The Role of Intersensory Redundancy,” Developmental Psychology 43 (2007): 238–252, 10.1037/0012-1649.43.1.238. 17201522 PMC2704007

[76] Chandrasekaran C, Trubanova A, Stillittano S, , “The Natural Statistics of Audiovisual Speech,” PLOS Computational Biology 5 (2009): e1000436, 10.1371/journal.pcbi.1000436. 19609344 PMC2700967

[77] Green JR, Nip ISB, Wilson EM, , “Lip Movement Exaggerations During Infant-Directed Speech,” Journal of Speech, Language, and Hearing Research 53 (2010): 1529–1542, 10.1044/1092-4388(2010/09-0005). PMC3548446 20699342

[78] Kitamura C, Guellaï B, and Kim J, “Motherese by Eye and Ear: Infants Perceive Visual Prosody in Point-Line Displays of Talking Heads,” PLoS ONE 9 (2014): e111467, 10.1371/journal.pone.0111467. 25353978 PMC4213016

[79] Rolf M, Hanheide M, and Rohlfing KJ, “Attention via Synchrony: Making Use of Multimodal Cues in Social Learning,” IEEE Transactions on Autonomous Mental Development 1 (2009): 55–67, 10.1109/TAMD.2009.2021091.

[80] Smith NA and Strader HL, “Infant-Directed Visual Prosody: Mothers’ Head Movements and Speech Acoustics,” Interaction Studies 15 (2014): 38–54, 10.1075/is.15.1.02smi. 25242907 PMC4166504

[81] Trainor LJ, Austin CM, and Desjardins RN, “Is Infant-Directed Speech Prosody a Result of the Vocal Expression of Emotion?,” Psychological Science 11 (2000): 188–195, 10.1111/1467-9280.00240. 11273402

[82] Tartter VC, “Happy Talk: Perceptual and Acoustic Effects of Smiling on Speech,” Perception & Psychophysics 27 (1980): 24–27, 10.3758/BF03199901. 7367197

[83] Gogate L, Bahrick LE, and Watson JD, “A Study of Multimodal Motherese: The Role of Temporal Synchrony Between Verbal Labels and Gestures,” Child Development 71 (2000): 878–894, 10.1111/1467-8624.00197. 11016554

[84] Gogate L, Maganti M, and Bahrick LE, “Cross-Cultural Evidence for Multimodal Motherese: Asian Indian Mothers’ Adaptive Use of Synchronous Words and Gestures,” Journal of Experimental Child Psychology 129 (2015): 110–126, 10.1016/j.jecp.2014.09.002. 25285369 PMC4252564

[85] Meyer M, Hard B, Brand RJ, , “Acoustic Packaging: Maternal Speech and Action Synchrony,” IEEE Transactions on Autonomous Mental Development 3 (2011): 154–162, 10.1109/TAMD.2010.2103941.

[86] Nomikou I and Rohlfing KJ, “Language Does Something: Body Action and Language in Maternal Input to Three-Month-Olds,” IEEE Transactions on Autonomous Mental Development 3 (2011): 113–128, 10.1109/TAMD.2011.2140113.

[87] de Villiers Rader N and Zukow-Goldring P, “How the Hands Control Attention During Early Word Learning,” Gesture 10 (2010): 202–221, 10.1075/gest.10.2-3.05rad.

[88] Suanda SH, Smith LB, and Yu C, “The Multisensory Nature of Verbal Discourse in Parent–Toddler Interactions,” Developmental Neuropsychology 41 (2016): 324–341, 10.1080/87565641.2016.1256403. 28128992 PMC7263485

[89] Brand RJ, Baldwin DA, and Ashburn LA, “Evidence for “motionese”: Modifications in Mothers’ Infant-Directed Action,” Developmental Science 5 (2002): 72–83, 10.1111/1467-7687.00211.

[90] Brand RJ, Hollenbeck E, and Kominsky JF, “Mothers’ Infant-Directed Gaze During Object Demonstration Highlights Action Boundaries and Goals,” IEEE Transactions on Autonomous Mental Development 5 (2013): 192–201, 10.1109/TAMD.2013.2273057.

[91] Iverson JM, Capirci O, Longobardi E, , “Gesturing in Mother-Child Interactions,” Cognitive Development 14 (1999): 57–75, 10.1016/S0885-2014(99)80018-5.

[92] Abu-Zhaya R, Seidl A, and Cristia A, “Multimodal Infant-Directed Communication: How Caregivers Combine Tactile and Linguistic Cues,” Journal of Child Language 1 (2016): 1–29, 10.1017/S0305000916000416. 27573414

[93] Tincoff R, Seidl A, and Buckley L, “Feeling the Way to Words: Parents’ Speech and Touch Cues Highlight Word-To-World Mappings of Body Parts,” Language Learning and Development 15 (2019): 103–125, 10.1080/15475441.2018.1533472.

[94] Lew-Williams C, Ferguson B, Abu-Zhaya R, , “Social Touch Interacts With Infants’ Learning of Auditory Patterns,” Developmental Cognitive Neuroscience 35 (2019): 66–74. 29051028 10.1016/j.dcn.2017.09.006 PMC5876072

[95] Seidl A, Tincoff R, Baker C, , “Why the Body Comes First: Effects of Experimenter Touch on Infants’ Word Finding,” Developmental Science 18 (2015): 155–164, 10.1111/desc.12182. 24734895

[96] Phillips-Silver J and Trainor LJ, “Feeling the Beat: Movement Influences Infant Rhythm Perception,” Science 308 (2005): 1430, 10.1126/science.1110922. 15933193

[97] Phillips-Silver J and Trainor LJ, “Hearing What the Body Feels: Auditory Encoding of Rhythmic Movement,” Cognition 105 (2007): 533–546, 10.1016/j.cognition.2006.11.006. 17196580

[98] Csibra G, “Recognizing Communicative Intentions in Infancy,” Mind & Language 25 (2010): 141–168, 10.1111/j.1468-0017.2009.01384.x.

[99] Csibra G and Gergely G, “Natural Pedagogy,” Trends in Cognitive Sciences 13 (2009): 148–153, 10.1016/j.tics.2009.01.005. 19285912

[100] Parise E and Csibra G, “Neural Responses to Multimodal Ostensive Signals in 5-Month-Old Infants,” PLoS ONE 8 (2013): e72360, 10.1371/journal.pone.0072360. 23977289 PMC3747163

[101] Bánki A, Köster M, Cichy RM, , “Communicative Signals During Joint Attention Promote Neural Processes of Infants and Caregivers,” Developmental Cognitive Neuroscience 65 (2024): 101321, 10.1016/j.dcn.2023.101321. 38061133 PMC10754706

[102] Leong V, Byrne E, Clackson K, , “Speaker Gaze Increases Information Coupling Between Infant and Adult Brains,” Proceedings of the National Academy of Sciences 114 (2017): 13290–13295, 10.1073/pnas.1702493114. PMC5740679 29183980

[103] Fernald A and Morikawa H, “Common Themes and Cultural Variations in Japanese and American Mothers’ Speech to Infants,” Child Development 64 (1993): 637–656, 10.2307/1131208. 8339686

[104] Hills T, “The Company That Words Keep: Comparing the Statistical Structure of Child- Versus Adult-Directed Language,” Journal of Child Language 40 (2013): 586–604, 10.1017/S0305000912000165. 22584041

[105] Lester NA, Moran S, Küntay AC, , “Detecting Structured Repetition in Child-Surrounding Speech: Evidence From Maximally Diverse Languages,” Cognition 221 (2022): 104986, 10.1016/j.cognition.2021.104986. 34953269

[106] Snow CE, “Mothers’ Speech to Children Learning Language,” Child Development 43 (1972): 549–565, 10.2307/1127555.

[107] Stern DN, Spieker S, Barnett RK, , “The Prosody of Maternal Speech: Infant Age and Context Related Changes*,” Journal of Child Language 10 (1983): 1–15, 10.1017/S0305000900005092. 6841483

[108] Tal S, Grossman E, and Arnon I, Infant-Directed Speech Becomes Less Redundant as Infants Grow: Implications for Language Learning. (2021), 10.31234/osf.io/bgtzd. 38810427

[109] Aslin RN and Newport EL, “Statistical Learning: From Acquiring Specific Items to Forming General Rules,” Current Directions in Psychological Science 21 (2012): 170–176, 10.1177/0963721412436806. 24000273 PMC3758750

[110] Saffran JR and Kirkham NZ, “Infant Statistical Learning,” Annual Review of Psychology 69 (2018): 181–203, 10.1146/annurev-psych-122216-011805. PMC5754249 28793812

[111] Schwab JF and Lew-Williams C, “Repetition Across Successive Sentences Facilitates Young Children’s Word Learning,” Developmental Psychology 52 (2016): 879–886, 10.1037/dev0000125. 27148781 PMC5651173

[112] Cox C, Bergmann C, Fowler E, , “A Systematic Review and Bayesian Meta-Analysis of the Acoustic Features of Infant-Directed Speech,” Nature Human Behaviour 7 (2023): 114–133, 10.1038/s41562-022-01452-1. 36192492

[113] Fernald A and Simon T, “Expanded Intonation Contours in Mothers’ Speech to Newborns,” Developmental Psychology 20 (1984): 104–113, 10.1037/0012-1649.20.1.104.

[114] Fernald A, “Approval and Disapproval: Infant Responsiveness to Vocal Affect in Familiar and Unfamiliar Languages,” Child Development 64 (1993): 657–674, 10.1111/j.1467-8624.1993.tb02934.x. 8339687

[115] Katz GS, Cohn JF, and Moore CA, “A Combination of Vocal f0 Dynamic and Summary Features Discriminates Between Three Pragmatic Categories of Infant-Directed Speech,” Child Development 67 (1996): 205–217, 10.1111/j.1467-8624.1996.tb01729.x. 8605829

[116] Soderstrom M, Blossom M, Foygel R, , “Acoustical Cues and Grammatical Units in Speech to Two Preverbal Infants,” Journal of Child Language 35 (2008): 869–902, 10.1017/S0305000908008763. 18838016

[117] Fernald A, “Intonation and Communicative Intent in Mothers’ Speech to Infants: Is the Melody the Message?,” Child Development 60 (1989): 1497–1510, 10.2307/1130938. 2612255

[118] Stern DN, Spieker S, and MacKain K, “Intonation Contours as Signals in Maternal Speech to Prelinguistic Infants,” Developmental Psychology 18 (1982): 727–735, 10.1037/0012-1649.18.5.727.

[119] Ratner B and Rooney B, “How Accessible Is the Lexicon in Motherese?,” in Approaches to Bootstrapping: Phonological, Lexical, Syntactic and Neurophysiological Aspects of Early Language Acquisition. Volume 1, ed. Weissenborn J, and Höhle B (John Benjamins Publishing Company, 2001), 71–78, 10.1075/lald.23.06ber.

[120] Genovese G, Spinelli M, Romero Lauro LJ, , “Infant-Directed Speech as a Simplified but Not Simple Register: A Longitudinal Study of Lexical and Syntactic Features,” Journal of Child Language 47 (2020): 22–44, 10.1017/S0305000919000643. 31663485

[121] Phillips JR, “Syntax and Vocabulary of Mothers’ Speech to Young Children: Age and Sex Comparisons,” Child Development 44 (1973): 182–185, 10.2307/1127699.

[122] Martin A, Igarashi Y, Jincho N, , “Utterances in Infant-Directed Speech Are Shorter, Not Slower,” Cognition 156 (2016): 52–59, 10.1016/j.cognition.2016.07.015. 27513869

[123] Henning A, Striano T, and Lieven EVM, “Maternal Speech to Infants at 1 and 3 Months of Age,” Infant Behavior and Development 28 (2005): 519–536, 10.1016/j.infbeh.2005.06.001.

[124] de la Cruz-Pavía I, Gervain J, Vatikiotis-Bateson E, , “Coverbal Speech Gestures Signal Phrase Boundaries: A Production Study of Japanese and English Infant- and Adult-Directed Speech,” Language Acquisition 27 (2020): 160–186, 10.1080/10489223.2019.1659276.

[125] Masataka N, “Motherese in a Signed Language,” Infant Behavior & Development 15 (1992): 453–460, 10.1016/0163-6383(92)80013-K.

[126] Chong SCF, Werker J, Russell J, , “Three Facial Expressions Mothers Direct to Their Infants,” Infant and Child Development 12 (2003): 211–232, 10.1002/icd.286.

[127] Shepard K, Spence M, and Sasson N, “Distinct Facial Characteristics Differentiate Communicative Intent of Infant-Directed Speech,” Infant and Child Development 21 (2012): 555–578, 10.1002/icd.1757.

[128] Brand RJ, Shallcross W, Sabatos M, , “Fine-Grained Analysis of Motionese: Eye Gaze, Object Exchanges, and Action Units in Infant-Versus Adult-Directed Action,” Infancy 11 (2007): 203–214, 10.1111/j.1532-7078.2007.tb00223.x.

[129] van Schaik JE, Meyer M, van Ham CR, , “Motion Tracking of Parents’ Infant- Versus Adult-Directed Actions Reveals General and Action-Specific Modulations,” Developmental Science 23 (2020): e12869, 10.1111/desc.12869. 31132212 PMC6916206

[130] Alviar C, Sahoo M, Edwards LA, , “Infant-Directed Song Potentiates Infants’ Selective Attention to Adults’ Mouths Over the First Year of Life,” Developmental Science 26 (2022): e13359, 10.1111/desc.13359. PMC10276172 36527322

[131] Trehub SE, Plantinga J, and Russo FA, “Maternal Vocal Interactions With Infants: Reciprocal Visual Influences,” Social Development 25 (2016): 665–683, 10.1111/sode.12164.

[132] Labendzki P, Goupil L, and Wass S, “Temporal Patterns in the Complexity of Child-Directed Song Lyrics Reflect Their Functions,” Communications Psychology 3 (2025): 48, 10.1038/s44271-025-00219-4. 40128378 PMC11933259

[133] Hansen M, “Children’s Rhymes Accompanied by Gestures,” Western Folklore 7 (1948): 50–53, 10.2307/1496690.

[134] Kragness HE, Johnson EK, and Cirelli LK, “The Song, Not the Singer: Infants Prefer to Listen to Familiar Songs, Regardless of Singer Identity,” Developmental Science 25 (2022): e13149, 10.1111/desc.13149. 34241934

[135] Fantz RL, “Visual Experience in Infants: Decreased Attention to Familiar Patterns Relative to Novel Ones,” Science 146 (1964): 668–670. 14191712 10.1126/science.146.3644.668

[136] Forest TA, McCormick SA, Davel L, , “Early Caregiver Predictability Shapes Neural Indices of Statistical Learning Later in Infancy,” Developmental Science 28 (2025): e13570, 10.1111/desc.13570. 39352772

[137] Carretero S, Español S, Rodríguez FG, , “Infant-Directed Improvised Performances, Protoconversations, and Action Songs During the First Year of Life,” in Moving and Interacting in Infancy and Early Childhood: An Embodied, Intersubjective, and Multimodal Approach to the Interpersonal World, ed. Español S, Martínez M, and Rodríguez FG (Springer International Publishing, 2022), 57–89, 10.1007/978-3-031-08923-7_3.

[138] Cirelli LK and Trehub SE, “Familiar Songs Reduce Infant Distress,” Developmental Psychology 56 (2020): 861–868, 10.1037/dev0000917. 32162936

[139] Dou A and Cirelli LK, “The Active Infant’s Developing Role in Musical Interactions: Insights From an Online Parent Questionnaire,” Infancy 30 (2025): e12648, 10.1111/infa.12648. 39780293 PMC11711306

[140] Custodero LA, “Singing Practices in 10 Families With Young Children,” Journal of Research in Music Education 54 (2006): 37–56, 10.2307/3653454.

[141] Ilari B, “Rhythmic Engagement with Music in Early Childhood: A Replication and Extension,” Journal of Research in Music Education 62 (2015): 332–343, 10.1177/0022429414555984.

[142] Marsh K and Young S, “Musical Play,” in The Child as Musician: A Handbook of Musical Development (Oxford University Press, 2006).

[143] Zentner M and Eerola T, “Rhythmic Engagement With Music in Infancy,” Proceedings of the National Academy of Sciences 107 (2010): 5768–5773, 10.1073/pnas.1000121107. PMC2851927 20231438

[144] Smith NA and Trainor LJ, “Infant-Directed Speech Is Modulated by Infant Feedback,” Infancy 13 (2008): 410–420, 10.1080/15250000802188719.

[145] Trainor LJ, Clark ED, Huntley A, , “The Acoustic Basis of Preferences for Infant-Directed Singing,” Infant Behavior and Development 20 (1997): 383–396, 10.1016/S0163-6383(97)90009-6.

[146] de l’Etoile SK, Behura S, and Zopluoglu C, “Acoustic Parameters of Infant-Directed Singing in Mothers of Infants With Down Syndrome,” Infant Behavior and Development 49 (2017): 151–160, 10.1016/j.infbeh.2017.09.001. 28934613

[147] Rock AM, Trainor LJ, and Addison TL, “Distinctive Messages in Infant-Directed Lullabies and Play Songs,” Developmental Psychology 35 (1999): 527–534, 10.1037//0012-1649.35.2.527. 10082023

[148] Delavenne A, Gratier M, and Devouche E, “Expressive Timing in Infant-Directed Singing Between 3 and 6 Months,” Infant Behavior and Development 36 (2013): 1–13, 10.1016/j.infbeh.2012.10.004. 23261784

[149] Bergeson T and Trehub S, “Mothers’ Singing to Infants and Preschool Children,” Infant Behavior and Development 22 (1999): 51–64, 10.1016/S0163-6383(99)80005-8.

[150] Fausey CM, Jayaraman S, and Smith LB, “From Faces to Hands: Changing Visual Input in the First Two Years,” Cognition 152 (2016): 101–107, 10.1016/j.cognition.2016.03.005. 27043744 PMC4856551

[151] Elmlinger SL, Schwade JA, Vollmer L, , “Learning How to Learn From Social Feedback: The Origins of Early Vocal Development,” Developmental Science 26 (2023): e13296, 10.1111/desc.13296. 35737680

[152] Zhang VH, Elmlinger SL, Albert RR, , “Caregiver Reactions to Babbling Organize Turn-Taking Interactions: Facilitative Effects of Vocal Versus Non-Vocal Responses,” Infancy 29 (2024): 525–549, 10.1111/infa.12596. 38696120 PMC11655154

[153] Kaplan BE, Kasaba I, Rachwani J, , “How Mothers Help Children Learn to Use Everyday Objects,” Developmental Psychobiology 65 (2023): e22435, 10.1002/dev.22435. 38010304 PMC12812014

[154] Deckner DF, Adamson LB, and Bakeman R, “Rhythm in Mother-Infant Interactions,” Infancy 4 (2003): 201–217, 10.1207/S15327078IN0402_03.

[155] Moreno-Núñez A, Murillo E, Casla M, , “The Multimodality of Infant’s Rhythmic Movements as a Modulator of the Interaction With Their Caregivers,” Infant Behavior and Development 65 (2021): 101645, 10.1016/j.infbeh.2021.101645. 34536806

[156] Cordaro DT, Sun R, Keltner D, , “Universals and Cultural Variations in 22 Emotional Expressions Across Five Cultures,” Emotion (Washington, D.C.) 18 (2018): 75–93, 10.1037/emo0000302. 28604039

[157] Marsh AA, Elfenbein HA, and Ambady N, “Nonverbal “Accents”,” Psychological Science 14 (2003): 373–376, 10.1111/1467-9280.24461. 12807413

[158] Kita S, Cross-Cultural Variation of Speech-Accompanying Gesture: A Review (Psychology Press, 2009).

[159] Boyne AS, Alviar C, and Lense M, “Parental Social and Musical Characteristics, the Home Music Environment, and Child Language Development in Infancy,” Infancy 30 (2025): e70008, 10.1111/infa.70008. 40022665 PMC12015385

[160] Yasinnik Y, Renwick M, and Shattuck-Hufnagel S, “The Timing of Speech-Accompanying Gestures With Respect to Prosody,” Journal of the Acoustical Society of America 115 (2004): 2397, 10.1121/1.4780717.

[161] Mehr SA, Singh M, York H, , “Form and Function in Human Song,” Current Biology 28 (2018): 356–368, 10.1016/j.cub.2017.12.042. 29395919 PMC5805477

[162] Trehub SE, Unyk AM, and Trainor LJ, “Adults Identify Infant-Directed Music Across Cultures,” Infant Behavior and Development 16 (1993): 193–211, 10.1016/0163-6383(93)80017-3.

[163] Singh M and Hill K, “Loss of Dance and Infant-Directed Song Among the Northern Aché,” Current Biology 35 (2025): 2444–2447, 10.1016/j.cub.2025.04.018. 40306280

[164] Trainor LJ and Cirelli L, “Rhythm and Interpersonal Synchrony in Early Social Development,” Annals of the New York Academy of Sciences 1337 (2015): 45–52, 10.1111/nyas.12649. 25773616

[165] de l’Etoile SK and Leider CN, “Acoustic Parameters of Infant-Directed Singing in Mothers With Depressive Symptoms,” Infant Behavior and Development 34 (2011): 248–256, 10.1016/j.infbeh.2010.12.013. 21255845

[166] Jaeger J, Borod JC, and Peselow E, “Facial Expression of Positive and Negative Emotions in Patients With Unipolar Depression,” Journal of Affective Disorders 11 (1986): 43–50, 10.1016/0165-0327(86)90058-3. 2944927

[167] Lam-Cassettari C and Kohlhoff J, “Effect of Maternal Depression on Infant-Directed Speech to Prelinguistic Infants: Implications for Language Development,” PLoS ONE 15 (2020): e0236787, 10.1371/journal.pone.0236787. 32730322 PMC7392317

[168] Warlaumont AS, Richards JA, Gilkerson J, , “A Social Feedback Loop for Speech Development and Its Reduction in Autism,” Psychological Science 25 (2014): 1314–1324, 10.1177/0956797614531023. 24840717 PMC4237681

[169] Fancourt D and Perkins R, “Associations Between Singing to Babies and Symptoms of Postnatal Depression, Wellbeing, Self-Esteem and Mother-Infant Bond,” Public Health 145 (2017): 149–152, 10.1016/j.puhe.2017.01.016. 28359384

[170] Lense M and Camarata S, “PRESS-Play: Musical Engagement as a Motivating Platform for Social Interaction and Social Play in Young Children With ASD,” Music & Science 3 (2020): 2059204320933080, 10.1177/2059204320933080. PMC7440205 32832103

[171] Dalla Bella S, Białuńska A, and Sowiński J, “Why Movement Is Captured by Music, but Less by Speech: Role of Temporal Regularity,” PLoS ONE 8 (2013): e71945, 10.1371/journal.pone.0071945. 23936534 PMC3732235

[172] Khalfa S, Roy M, Rainville P, , “Role of Tempo Entrainment in Psychophysiological Differentiation of Happy and Sad Music?,” International Journal of Psychophysiology 68 (2008): 17–26, 10.1016/j.ijpsycho.2007.12.001. 18234381

[173] London J, Hearing in Time: Psychological Aspects of Musical Meter (Oxford University Press, 2012).

